# Pollution Risk Assessment and Sources Analysis of Heavy Metal in Soil from Bamboo Shoots

**DOI:** 10.3390/ijerph192214806

**Published:** 2022-11-10

**Authors:** Zhihong Wang, Yingle Chen, Song Wang, Yujuan Yu, Wenyan Huang, Qiaolin Xu, Lei Zeng

**Affiliations:** Guangdong Provincial Key Laboratory of Silviculture, Protection and Utilization, Guangdong Academy of Forestry, Guangzhou 510520, China

**Keywords:** bamboo shoot soil, heavy metal, pollution risk assessment, source analysis, Guangdong Province, multivariate statistical

## Abstract

In order to investigate the pollution situation and sources analysis of heavy metals in bamboo shoot soil in Guangdong Province, a total of 175 soil samples were collected at 46 sites. Atomic fluorescence spectrophotometer and inductively coupled plasma mass spectrometry were used to determine the content of five heavy metals: lead (Pb), cadmium (Cd), arsenic (As), mercury (Hg), and chromium (Cr). In addition, the soil environmental quality was evaluated through different index methods, including single-factor pollution, Nemeiro comprehensive pollution, geoaccumulation, and potential ecological risk. Furthermore, the correlation coefficients were also discussed. The results showed that the soils collected were acidic or slight alkaline. The maximum content of Pb and As from some areas exceeded the standard limit value. The coefficient of variation value from six areas exceeded 100%. The index method mentioned above confirmed that the soil within study areas was divided into three pollution levels: no, slightly, and mild. Additionally, there was a very significant correlation between pH and Pb, Hg; the correlation between heavy metal As and Pb, Cr also reached a very significant level. The principal component analysis results show that PC1 accounts for 39.60% of the total variance, which includes Pb, Cd, and As. PC2 mainly includes Hg and Cr.

## 1. Introduction

Bamboo is a perennial evergreen plant belonging to the subfamily Bambooaceae of the Poaceae family [1,2]. It is a versatile and highly renewable woody stem perennial herb found primarily in moist deciduous, semi-evergreen, tropical, subtropical, and temperate forest areas [3]. More than 1250 species belonging to 75 genera of bamboo have been reported around the world [4]. Bamboo shoots have great potential as a food resource and are considered “the best vegetarian food” [5]. They are rich in essential nutrients such as dietary fiber, protein, carotene, vitamins, selenium, potassium, calcium, phosphorus, iron, and are a good source of nutrients high in fiber and low in fat [1,2,6]. They are not only a storehouse of nutritional elements, but also contain some important antioxidants and other functional components, such as polyphenols, flavonoids, etc., which help prevent metabolic disorders, cardiovascular diseases, cancer, diabetes, hypertension, and obesity [7,8,9,10,11].

China has abundant bamboo resources, and it ranks first in the world in terms of type, area, stock, and output of bamboo and bamboo shoots. As a traditional forest vegetable, edible bamboo shoots are one of the commonly collected, consumed, and sold nutritious vegetables among rural and urban areas of southern China. Guangdong Province is an important producing area of bamboo shoots in China, and the production areas are mainly concentrated in Zhaoqing, Jieyang, Qingyuan, Shaoguan, and Meizhou, etc. The Guangdong bamboo shoots are of good quality and are very popular among people, and related products have been exported to many countries and regions [6]. However, with the increase in demand for bamboo shoots, the production mode of bamboo shoots has changed from the traditional management mode to the intensive management mode. The growth process of bamboo shoots needs to take measures, such as fertilization and pesticide application, which may cause problems, such as soil heavy metal pollution [12,13,14,15]. Further, soil pollution by heavy metals was already a global problem and is a concern. According to statistics, more than 10 million soil sites are contaminated worldwide, and more than 50% of these sites are polluted by heavy metals [16]. In addition, approximately 1/6 of the agricultural and forestry land in China may currently suffer from heavy metal pollution [17]. It is worth noting that soil is an important carrier for human survival, and the physical and chemical properties of soil are greatly affected by the natural geological background and human activities. Soil is a complex system composed of solid phases (soil minerals and organic matter) and fluid phases (soil water and soil air) [18]. Heavy metals are mostly precipitated on the surface of soil minerals in the form of oxides, hydroxides, carbonates, sulfides, and phosphates [19]. Heavy metals are a class of refractory pollutants with strong persistence and easy accumulation in living organisms, and have the characteristics of toxicity, refractory, irreversibility, ubiquity, and easy accumulation [20,21,22,23].

As we all know, heavy metals in soils come from a wide range of sources, mainly including weathered parent materials, the impact of mining, transportation emissions, smelting products, fertilizer application, and other industrial, agricultural, and commercial activities [24,25]. Based on the World Health Organization, the Ministry of Environmental Protection of the People’s Republic of China and the United States Environment Protection Agency, heavy metal contamination will affect soil quality and crop growth, and with the increase of their content, they will pose a serious threat to the ecosystem and human health through different exposure pathways [26]. Excessive accumulation of soil heavy metals in plants will destroy soil quality and affect the growth and metabolism of plants, and endanger water and the atmospheric environment, exacerbate global climate change, and affect sustainable social development. Above all, it may also accumulate in the human body through the food chain or other means, directly affecting the quality of related products and the health of consumers [27,28,29,30,31]. Therefore, it is meaningful to estimate the contamination level of soil to improve the soil environment and protect human health. Previous research results have shown that heavy metals (Pb, Cd, Hg, Cr) and metalloids (As) account for more than 82.0% of the soil contamination rate [32]. Many research studies have focused on pollution levels, health risk, and sources apportionment in single land use type, such as agricultural land, forest land, industrial land, and residential land [28,33,34,35,36]. In addition, atomic absorption spectrophotometer and inductively coupled plasma mass spectrometry are used for heavy metals content detection, while the single-factor pollution index method, Nemeiro comprehensive pollution index method, geoaccumulation index method, and the potential ecological risk index method were used for risk assessment [32]. Meanwhile, various mathematical models and statistical methods, such as enrichment factors and principal component analysis and so on, have also been used to analyze the source of soil pollution. Thus, soil heavy metal contamination has attracted global attention and is listed as the focus of pollution monitoring and control. In this study, Pb, Cd, As, Hg, and Cr are collectively named as heavy metals for the reason of simplification.

Based on the above considerations, heavy metals are closely related to soil environmental pollution, ensure food safety, and improve people’s health. There have been a few reports on heavy metal pollution risk assessment and source analysis of bamboo shoot soil in Guangdong province. Thus, a total of 175 bamboo shoot soil samples from representative bamboo shoot production areas were collected and analyzed. The objectives of this study were: (1) to investigate the pH value and heavy metal contents from different representative bamboo shoot planting areas; (2) to evaluate the ecological risk of soil heavy metals using single-factor pollution index, Nemerow pollution index, geoaccumulation index, potential risk assessment; and (3) to explore the relative influence of different sources on heavy metal pollution using correlation analysis and principal component analysis, which could provide a scientific basis and theoretical reference for high value utilization, contamination risk prevention, and industrialization development of bamboo shoots in Guangdong.

## 2. Methods and Materials

### 2.1. Sample Collection and Analysis

**Figure 1:**
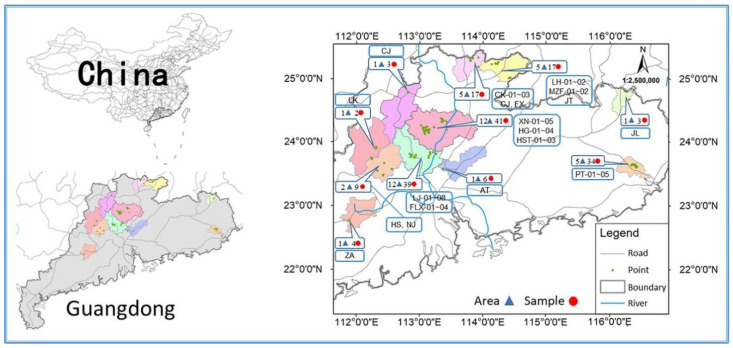
Location of the study area and sample point.

The soil samples were obtained from several large bamboo shoot growth bases in Guangdong Province in China, where the latitudes range from 22°45′57″ to 25°27′19″ and the longitudes from 111°52′32″ to 116°25′28″. In total, 175 soil samples were collected in 7 cities of different bamboo shoot production bases, and these samples were 46 subareas collected from 2020 to 2021. Detailed sample collection areas, sample numbers, and distribution points are presented in Figure 1. After removing impurities such as large particle size grave, weeds, and plant roots, etc., the subsurface soil (0–20 cm below the ground surface) was sampled from each soil sampling site. Approximately 1 kg of surface soil sample were collected from each site in the study area. According to the “S” shape uniform and random method, the soil of 5 sample points was collected, and then fully mixed. Then, an appropriate amount of the sample was put into a polyethylene film sealing bag and labeled, then stored at 4 °C for further analysis. At the same time as the soil was collected, the corresponding bamboo shoot samples were also collected. As with soil samples, bamboo shoots were packaged and labeled. Fresh bamboo shoot samples were transported back to the laboratory immediately, where they were shelled and shredded and stored in liquid nitrogen at −80 °C for further analysis. According to food safety standards of the People’s Republic of China (GB 5009.268-2016), the sample to be measured was homogenized and digested by microwave. The heavy metal (Pb, Cd, and As) content of the sample was then measured by inductively coupled plasma-mass spectrometry (ICP-MS).

The collected soil samples were placed in a dry and ventilated place to dry naturally, and then grounded in the laboratory using a ceramic rod mill and grinder model, respectively. The soil samples then passed through a 100-mesh sieve and were digested by mix acid (HCl-HNO_3_) solution for the analysis of heavy metal concentration in the soil samples. The pH of the soil sample was determined through a soil-to-water mixture (1:2.5, *w*/*v*) using the potentiometric method [37,38,39]. The total concentration of five heavy metal (Pb, Cd, As, Hg, and Cr), as the priority elements in the Chinese Soil Environmental Quality Risk Control Standard for Soil Contamination of Agricultural Land (GB15618-2018) [40], were of concern in this study. Additionally, the Pb, Cd, and Cr concentrations were determined by ICP-MS, according to the Environmental Protection Standards of the People’s Republic of China (HJ 803-2016) [41], and the detection limits were 10, 0.1, and 15 mg/kg, respectively. Further, the As and Hg content was measured using atomic fluorescence spectrometry according to the Ministry of Agriculture standards GB T22105.1-2008 [42] and GB T22105.2-2008 [43], and the detection limits were 0.002 and 0.05 mg/kg, respectively. It is worth noting that the mixed acid used in the former is hydrochloric acid and nitric acid mixed in a ratio of 3:1, while the latter needs to be diluted 1 time before use. By the above analysis method, the classification of heavy metal concentration in the soil was evaluated according to Standard (GB15618-2018).

### 2.2. Data Analysis

Microsoft Excel 2016 was used to collect the data and descriptive statistics. All data are presented as the means ± standard deviations (SD) and all statistical analyses were performed using SPSS 21.0 software. In addition, an analysis of variance was performed using Duncan’s test, where *p* < 0.05 was considered statistically significant. The box plot and histograms were plotted in origin 2021.

## 3. Results and Discussion

### 3.1. Soil Properties and Soil Heavy Metal Contents

**Figure 2:**
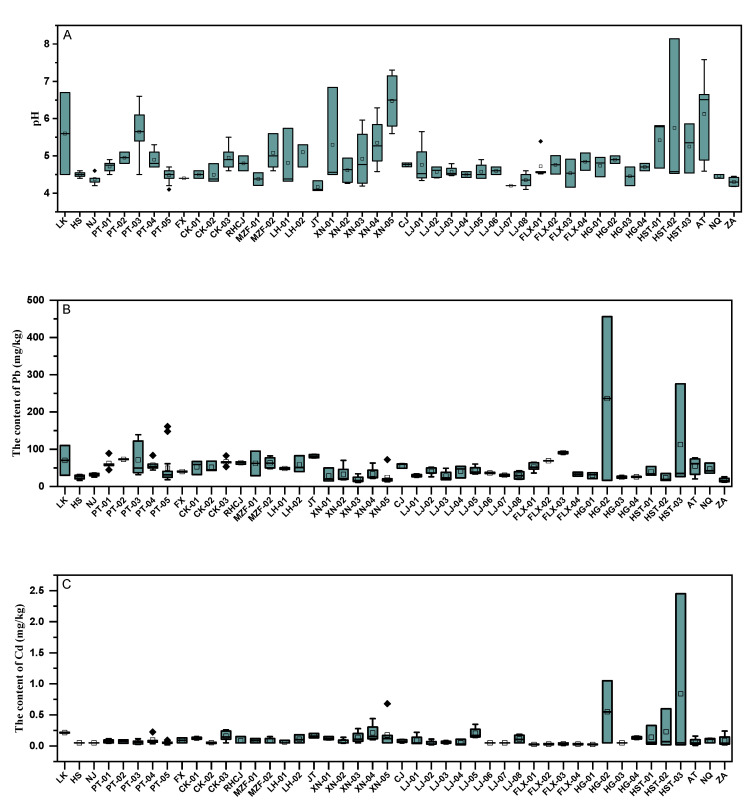

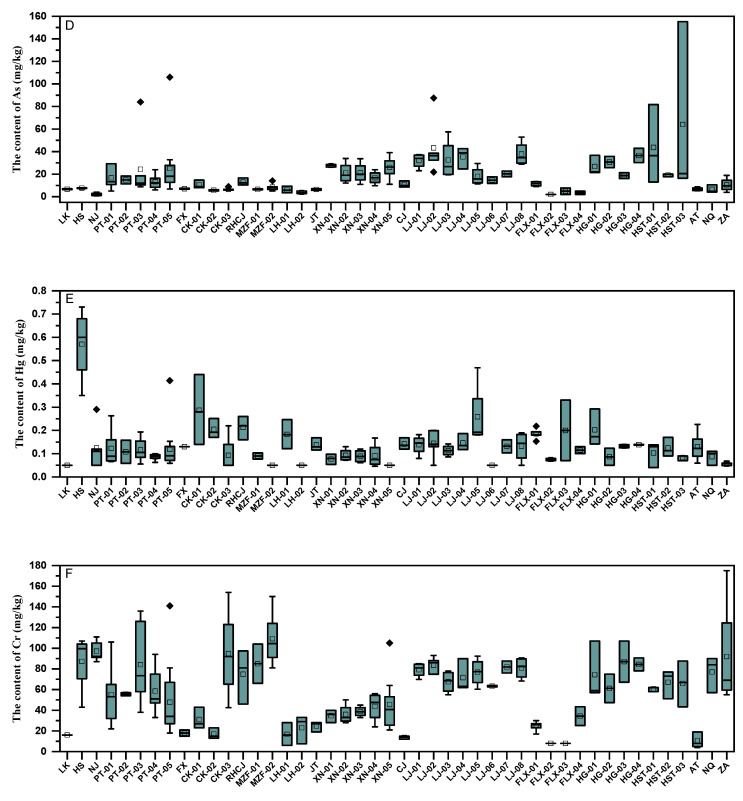
pH value (**A**) and heavy metal content of Pb (**B**), Cd (**C**), As (**D**), Hg (**E**), and Cr (**F**) from different bamboo shoot areas.

The statistical results and limit standard of soil heavy metals are presented in Table 1. The mean contents of Pb, Cd, As, Hg, and Cr were higher than soil background value [44], with a rate of 1.42, 3.24, 1.78, 1.73, and 1.33 times, respectively. The pH values in Bamboo shoots soils ranged from 4.07 to 8.14, indicating that cultivated land in this study area had weak acidity to weak alkalinity. As shown in Figure 2A, except for the XN-05 area (pH = 6.48) and AT area (pH = 6.12), the average soil pH of other bamboo shoot production bases was lower than 6.0, but the soil pH values at individual sampling points in the XN-5 and HST-02 regions were relatively high, with values higher than 7, but lower than 8.

General description statistics of the five heavy metals (Pb, Cd, As, Hg, and Cr) concentration in the study soil are provided in Figure 2B–F. The mean content of the studied elements followed the increasing order of Cr > Pb > As > Hg > Cd. For all bamboo shoot-producing soils, in terms of individual sampling points in different areas, the contents of Pb, Cd, As, and Cr exceeded the risk screening reference standard value to a certain extent. However, the average content of heavy metals Cr in each area did not exceed the risk screening value (150 mg/kg), and for other heavy metals, the average Pb content of PT-02, JT, FLX-03, HG-02, and HST-03 areas exceeded 70 mg/kg, and the average As content of HST-01 and HST-03 areas also exceeded the risk screening value (40 mg/kg), and the average Cd content of HG-02 and HST-03 areas was also higher than the risk screening value (0.3 mg/kg). Therefore, the pollution risk, grade assessment, and source of heavy metals such as lead, cadmium, and arsenic should be of particular concern.

The coefficient of variation (CV) usually reflects the difference in the distribution of heavy metal content in each sample. The value of the coefficient of variation is related to human activities. The coefficient of variation CV ≤ 10% indicates weak variability; 10% < CV < 100% means moderate variability; CV ≥ 100% refers to strong variability [45]. The calculation results of CVs are shown in Table 2. The CV value analysis results show that, compared with other heavy metals, 72% of the pH is lower than 10% in different regions, which belongs to the weak variation category. As for the CV values of Hg and Cr, 80.43% and 82.61% of the sample regions were moderate variability, respectively, and other regions were weak variability. However, the CV value analysis results of Pb, Cd, and As showed that in the PT-03 (As), HG-02 (Pb, Cd), HST-01 (Cd), HST-02 (Cd), HST-03 (Pb, Cd and As), and ZA (Cd) areas, the CV values of the corresponding heavy metals exceeded 100%. The high CVs of Pb, Cd, and As indicated that these metals in the study soil differed greatly with respect to different sample areas and were seriously impacted by human activities.

### 3.2. Pollution Assessment of Heavy Metals

#### 3.2.1. Evaluation of Pollution Index

The single factor pollution index (*P_i_*) is the ratio of the measured concentration of pollutants to their corresponding evaluation criteria. This method can only evaluate a single heavy metal factor and cannot reflect the pollution level caused by multiple pollution factors. The Nemerow comprehensive pollution index (*P_N_*) is a method for evaluating the pollution level by comprehensively analyzing the average and maximum values of the single-factor pollution index, which incorporates the minimum and maximum value of the different heavy metals concentration and can comprehensively evaluate the pollution caused by a variety of pollution factors [46,47,48]. *P_i_* and *P_N_* can be calculated as follows:

$$P_{i}=\frac{C_{i}}{S_{i}}$$

$$P_{N}=\sqrt{\frac{P_{i\;max}^{2}+P_{i\;ave}^{2}}{2}}$$

where *C_i_* is the measured heavy metal concentration in the individual soil sample and *S_i_* is the reference standard concentration value of each metal in soil. In this study, *S_i_* is the soil risk screening value for heavy metal contamination of agricultural land in China, GB 15618-2018. The subscripts *max* and *ave* denote the maximum and average values of the single factor pollution index, respectively. The contamination degree of soil sample can be classified based on *P_i_* and *P_N_*. Additionally, the classification is listed in Table 3.

**Figure 3:**
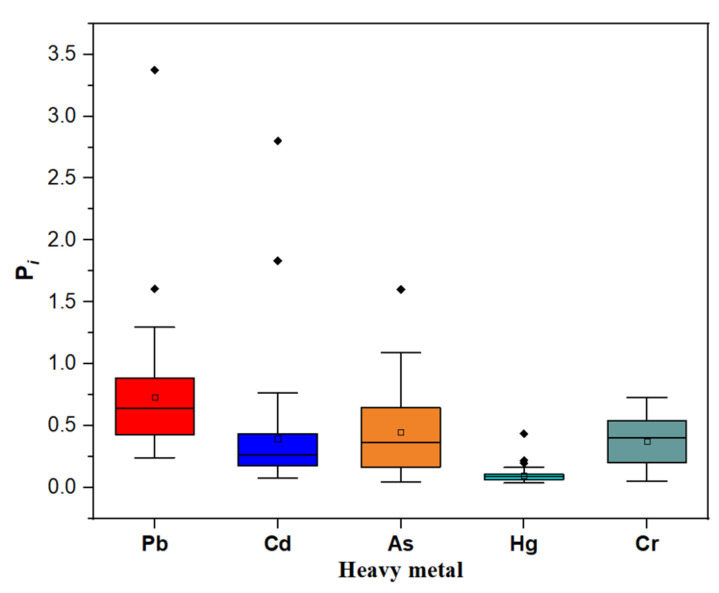
The single pollution index (*P_i_*) of five heavy metals in the subsurface soil of the study area.

The analysis results of the single pollution index are shown in Figure 3. The results show the single pollution index (*P_i_*) values of heavy metals in the subsurface soil of the study area. The *P_i_* of Pb from LK, PT-02, PT-03, JT, FLX-03, HST-03 areas were more than 1.0, which were in the slight accumulation level, and the *P_i_* of Pb in the HG-02 area was 3.37, reaching the medium accumulation level. For Cd element, the *P_i_* of HG-02 was 1.83 and the *P_i_* of HST-03 researched 2.8, which belong to the slight and mild accumulation level, respectively. The *P_i_* value of As in LJ-02, HST-01, and HST-03 areas were 1.08, 1.09, and 1.60, respectively, which were also in the slight accumulation levels. While the *P_i_* values of Hg, Cr were less than 1, which are in the no accumulation level. The average value of the single pollution index of heavy metals is Pb > As > Cd > Cr > Hg, indicating that among the five heavy metals, only the concentrations of Pb, Cd, and As in the individual study area were above the reference threshold value. These results show that Pb was the most polluting metal in the research areas, followed by Cd and As.

**Figure 4:**
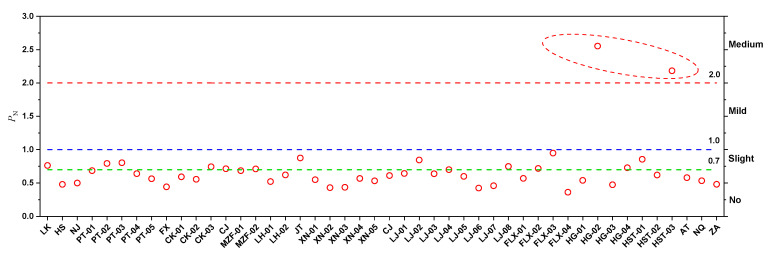
The Nemerow comprehensive pollution index (*P_N_*) of five heavy metals in the subsurface soil of the study area.

The Nemerow integrated pollution index (*P_N_*) analysis showed that the *P_N_* value of the study soils areas varied from 0.361 to 2.55 (See Figure 4), and the proportion of soil samples classified as no accumulation, slight accumulation, and medium accumulation were 67.37%, 28.26%, and 4.35%, respectively. These results further confirm that many soil samples in the study areas were in the safe level from the Nemerow integrated pollution index method. Since heavy metals can accumulate in edible parts of plants through the food chain, the heavy metal content and health risk of various products in this area should receive greater attention.

#### 3.2.2. Geoaccumulation Index

The index of geoaccumulation (*I_geo_*) was first introduced by Muller (1969) [49], which can be used to evaluate the relationship between the average concentration of heavy metal in the sample area and the total concentration of heavy metal in the corresponding sample, and it is often adopted to quantitatively describe the contamination level of the samples corresponding to the entire investigation area. This is because it not only reflects the influence of background values caused by natural geological processes, but also fully pays attention to the influence of human activities on heavy metal pollution [50].

$$I_{geo}=log_{2}\left(\frac{C_{i}}{1.5B_{i}}\right)$$

where *C_i_* is the measured heavy metal concentration in the soil sample, *B_i_* is background value of the corresponding heavy metal in the study area. The contamination level of the heavy metal could be evaluated by the *I_geo_* value, and its pollution grade standard is as follows: no contamination (*I_geo_* ≤ 0), litter contamination (0 < *I_geo_* ≤ 1), medium contamination (1 < *I_geo_* ≤ 2), medium contamination (2 < *I_geo_* ≤ 3), heavy contamination (3 < *I_geo_* ≤ 4), heavy contamination (4 < *I_geo_* ≤ 5), very heavy contamination (*I_geo_* > 5).

**Figure 5:**
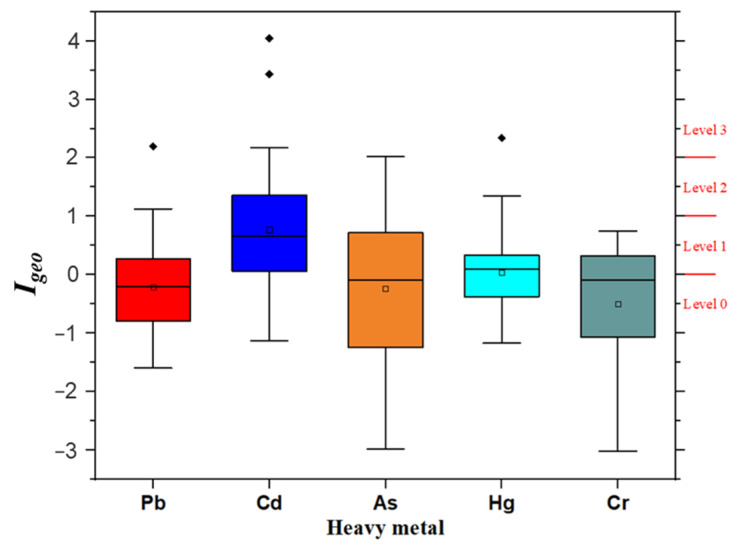
The *I_geo_* levels of five heavy metals in bamboo shoots soil of the research areas.

The geoaccumulation index (*I_geo_*) is a common index used to characterize the degree of heavy metal enrichment in sediments and soils. The geoaccumulation index of five heavy metals in all soil samples was analyzed, and the results are shown in Figure 5. The mean values of the *I_geo_* of Pb, Cd, As, Hg, and Cr were −0.2108, 0.7718, −0.2306, 0.0453, and −0.4986, respectively. The *I_geo_* of Pb showed that the values in the HG-02 and HST-03 areas were at level 3 (2.20) and level 2 (1.12), respectively, and the proportion of *I_geo_* of Pb at level 0 and level 1 was 95.65%. The *I_geo_* of Cd of all soil samples below level 0, level 1, level 2, and level 3 were 21.74%, 43.48%, 21.74%, and 13.04%, respectively, and that in level 1 was the largest, showing that there was light contamination of bamboo shoots in the soil study area. However, in both HG-02 and HST-03 areas, the *I_geo_* value of Cd exceeded 3.0. The analysis results of the As *I_geo_* value revealed that only the value of HST-03 areas was higher than 2.0 (level 03), and the proportion order of other different pollution grades was as follows: level 0 (52.17%) > level 1 (30.43%) > level 2 (15.22%). The results for *I_geo_* of Hg showed that the regions at level 2 and 3 were CK-01 (1.35), LJ-05 (1.20), and HS (2.34), while other areas were classified as No and litter contamination, accounting for 93.48% of all soil sample areas. The proportion of Cr in the soil sample points below level 0 and level 1 were 54.35% and 45.65%, respectively, indicating that there was little chromium contamination of soil in the research area.

#### 3.2.3. Evaluation of Ecological Risk Index

The ecological risk index (RI) was proposed by Hakanson (1980) [51] from the perspective of sedimentology and was mainly used to describe heavy metal concentration and its potential risk to the ecological system [45]. The RI value was calculated based on the following equation:

$$E_{r}^{i}=T_{r}^{i}\frac{C_{s}^{i}}{C_{n}^{i}}$$

$$\mathrm{RI}=\overset{n}{\underset{i=1}{\sum }}E_{r}^{i}$$

where $E_{r}^{i}$ is potential ecological hazard coefficients of single elements in Soil, which is mainly used to evaluate the pollution level of a certain element in the soil. $T_{r}^{i}$ is the toxicity response coefficient of heavy metal. To reflect the toxicity level of heavy metals and the sensitivity of the environment to heavy metal pollution, the toxicity response coefficient of different heavy metals: Pb = 5, Cd = 30, As = 10, Hg = 40, Cr = 2. $C_{s}^{i}$ is the measured heavy metal concentration in the study, $C_{n}^{i}$ is the evaluation standard/reference value of corresponding heavy metals in the study area. The value of RI can describe the synergistic effects of various elements, toxicity levels, contamination concentrations, and environmental sensitivity on heavy metal contamination, and can also comprehensively reflect the impact potential of heavy metals on the ecological environment. Based on the accuracy and universality of Hakanson’s potential ecological risk assessment results, refer to the classification method proposed by Li et al. (2018) [52], (see Table 4). The potential ecological risk levels of heavy metals in soil of the study area could be reclassified according to their toxicity and species.

$E_{r}^{i}$ The potential ecological hazard index method not only considers the content of heavy metals, but also combines the ecological effects, environmental effects, and toxicology of heavy metals, and presents the potential ecological risks of heavy metals through intuitive quantitative values. It is one of the most common methods for evaluating soil heavy metal pollution and ecological risks.

The potential ecological risk index, RI, was adopted to evaluate soil heavy metal contamination. As shown in Table 5, in all the soil samples from 46 areas, the mean value of potential ecological hazard coefficient ($E_{r}^{i}$) for five metals is as follows: Cd > As > Hg > Pb > Cr. The potential ecological hazard coefficient of Cd in level 1 accounted for 95.65%, and that in level 2 accounted for 4.35%, indicating that the degree of ecological risk was at the light and medium level. The potential ecological hazard coefficients of Pb, As, Hg, and Cr were all less than 30, indicating that the degree of ecological risk was at a light level. The above study results showed that there was a risk of chromium contamination in the bamboo shoots soil samples of the different research areas.

The potential ecological risk index indicated that except for HG-02 and HST-03 bamboo shoots production areas, RI values of the soil samples from other areas were all less than 60, and it falls in the category of light ecological risk. Additionally, the RI values of HG-02 and HST-03 were 83.02 and 111.48, respectively, which belonged to medium ecological risk. It was worth noting that the potential ecological risk index of five heavy metals in all soil samples of the study area was 1146.02, which reached the extremely strong ecological risk degree. The potential ecological risk index values of the five heavy metals ranged from 34.26 to 548.78. Furthermore, average contribution rates of the $E_{r}^{i}$ values to the RI values of five heavy metals were as follows: Cd (47.89%) > As (18.06%) > Hg (16.39%) > Pb (14.67%) > Cr (2.99%).

According to the analysis results obtained, except for HG-02 and HST-03 regions, the potential ecological risk level of each bamboo shoots soil sampling area was low, and the possibility of heavy metal contamination was very low, but the superposition of all soil samples from different areas might lead to a very strong ecological risk to soil in the study area, and the ecological risk of chromium was the most serious.

### 3.3. Analysis of Heavy Metals in Bamboo Shoots and Soil

#### 3.3.1. Bioconcentration Factor

Bioconcentration factor (BCF) can reflect the ability of bamboo shoots to absorb and accumulate heavy metals from soil, and it is expressed by the ratio of heavy metal concentration in a part of bamboo shoots to that in corresponding soil samples [53,54]. The BCF value was calculated based on the following equation:

$$\mathrm{BCF}=\frac{C_{R}}{C_{S}}$$

where *C_R_* is the heavy metal concentration in the bamboo shoots. *C_s_* is the soil heavy metal concentration in the corresponding soil sample of the research area. The larger the value of BCF, the stronger the enrichment ability of bamboo shoot samples for heavy metals in soil.

**Figure 6:**
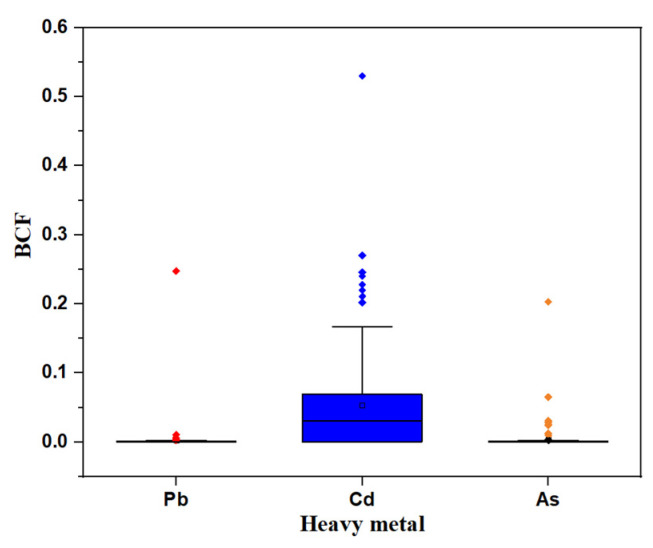
The bioconcentration factor of three heavy metals including Pb, Cd, and As.

The BCF value of different heavy metals in bamboo shoots was used to evaluate the accumulation degree, and it is shown in Figure 6. The average BCF values of three heavy metals in bamboo shoots were less than 0.1, the values of BCF of Pb and As were both less than 0.004, which were 0.0031 and 0.0039, respectively. The results elucidated that the accumulation ability of heavy metals in bamboo shoots was generally low. However, the BCF of Cd was 0.0534, which was higher than that of other heavy metals, and which also indicated that Cd was more easily accumulated in bamboo shoots in the study area. In addition, it was worth noting that the BCF values of Pb, Cd, and As heavy metals in bamboo shoots at individual sampling points were higher than 0.1, among which the BCF value of Cd reached 0.53, Pb and As were 0.2475 and 0.203, respectively. According to these results, it was speculated that the ability of plants to accumulate heavy metals may be affected by the external growth environment [55].

#### 3.3.2. Relationship between Bamboo Shoots and Soil

**Figure 7:**
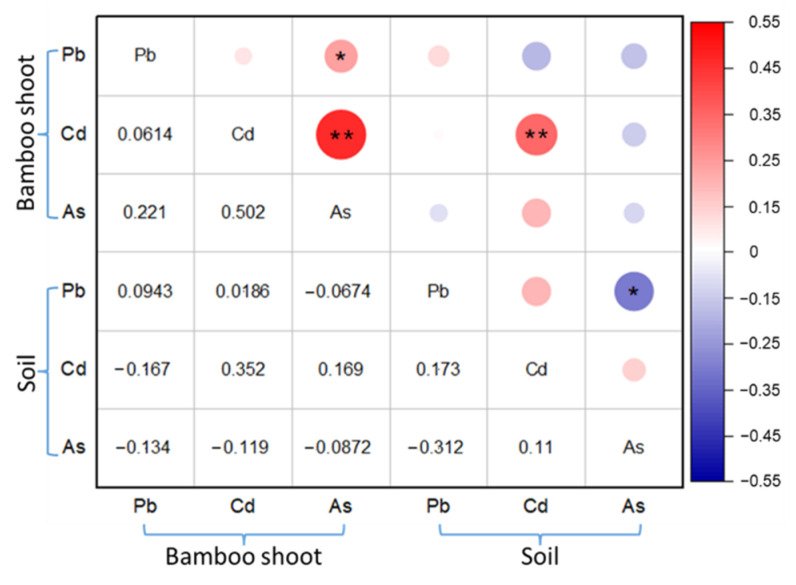
Correlation coefficient between the characteristic heavy metals from soil and bamboo shoots in the corresponding growth environment. ** significant at the 0.01 level; * significant at the 0.05 level.

The correlation analysis of bamboo shoot samples from bamboo shoot producing areas and their corresponding soil heavy metal contents, is shown in Figure 7. In the study area, except for the heavy metal Cd in bamboo shoot samples, which showed a very significant positive correlation with the corresponding soil Cd content (*p* < 0.01), the correlations between other heavy metals did not reach a significant level. The accumulation process of heavy metals from soil to plants was very complex, which may be controlled by the interaction between plants, soil, and metals. Besides soil, heavy metal residues in water or air might also be associated with heavy metal enrichment in plants. Based on the results of this study, it could be further speculated that soil might not be the main factor related to the accumulation of heavy metals in bamboo shoots, and the accumulation of heavy metals in bamboo shoots could be related to other factors. Although not significant, a negative correlation trend between the heavy metal As in soil and As content in bamboo shoots could still be observed. This means that within a certain concentration range, the As content in bamboo shoots decreased with the increase of As content in soil. This negative correlation between heavy metals in plants and soil might be at least partially attributable to the potential defense capacity of plants against heavy metal stress [55].

### 3.4. Multiple Bi-Variate Statistical Analysis

#### 3.4.1. Correlation Analysis

**Figure 8:**
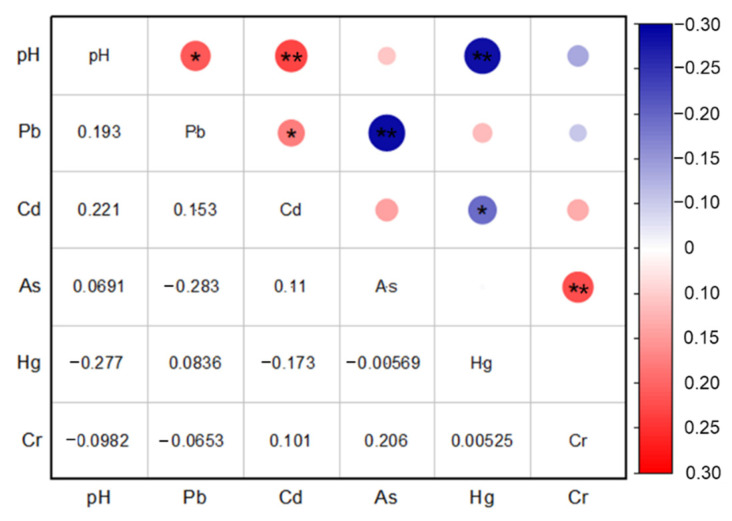
Correlation coefficient matrix of heavy metals in bamboo shoot soil. ** significant at the 0.01 level (2-tailed); * significant at the 0.05 level (2-tailed).

As shown in Figure 8, the results of the Spearman correlation coefficients for soil pH and the five heavy metals are presented. Through correlation coefficient analysis, interesting information about the source and diffusion process of heavy metals can be obtained. The significant positive correlation was observed between pH value and Pb (*p* < 0.05), pH value and Cd (*p* < 0.01), on the contrary, soil pH showed a high significant negative correlation with Hg (*p* < 0.01), indicating that the concentration of Cd and Pb had decreasing trends in the weak alkaline soil, but Hg had opposite trends. There was a significant negative correlation between Pb and As (*p* < 0.01), Cd and Hg (*p* < 0.05), and the correlation coefficients were −0.283 and −0.173, respectively. In addition, the positive coefficients of correlation between Pb and Cd were significant (*p* < 0.05), and As also showed a positive correlation with Cr (*p* < 0.01), indicating that these two heavy metals might also have a same source.

#### 3.4.2. Relationship of pH Value and Heavy Metal

It is well known that pH is one of the most important indicators affecting the content of heavy metals in soil. The forms of heavy metals in soil were free and complex. The increase in acidity makes the complexed heavy metals dissolve and become free. The free heavy metals are easily lost from the soil under the effect of rain erosion and plant enrichment, which may also be the main reason for the change of heavy metal content with the increase of soil acidity.

Generally, soil has a certain amount of negative charges, which is conducive to the adsorption of positively charged heavy metal ions such as Cu (II), Zn (II), and Cd (II) [56]. The adsorption capacity of oxygen anions is enhanced [57], and the adsorption capacity of positively charged heavy metal cations is weakened [58,59]. Therefore, it is necessary to study the correlation between soil pH and heavy metal content.

**Figure 9:**
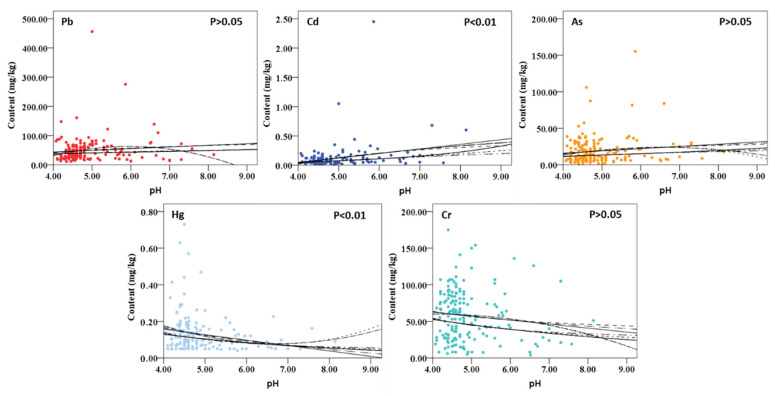
The relationship between heavy metals content and pH value of soil sample.

According to the data of soil pH and different heavy metal contents obtained in the experiment, 11 models (linear, logarithmic, reciprocal, quadratic, cubic, compound model, power, sigmoid, growth model, exponential model, and logistic distribution) were selected to perform regression analysis in order to explore the relationship between soil pH and heavy metal content. The model fitting results are shown in Figure 9. It can be found that the contents of heavy metals Pb, As, and Cr have no significant correlation with soil pH value (*p* > 0.05). However, there is a very significant correlation between soil pH value and heavy metal Cd and Hg content (*p* < 0.01). The results show that the pollution control and content prediction of heavy metals Cd and Hg can be achieved by the soil pH value.

Soil pH value not only affects the ionic composition of soil and various chemical reactions in soil, but also affects the bioavailability of soil heavy metals, the migration of heavy metals in the soil–plant system, and the passivation and remediation effect of heavy metal pollution, which is very important for plant growth. In addition, soil pH is an important chemical property of soil, and it is also a comprehensive reflection of other chemical properties of soil. It is related to the activities of soil microorganisms, the synthesis and decomposition of organic matter, the availability, transformation, and release of various nutrient elements, and the ability of soil to retain nutrients.

#### 3.4.3. Principal Component Analysis

Principal component analysis (PCA) is a method for concentrating and extracting the information of heavy metal pollutants in soil, so as to confirm the source of heavy metal contamination [60]. The Kaiser–Meyer–Olkin (KMO) measure and Bartlett’s test of sphericity were performed to analyze the suitability of the 175 soil samples from 46 production areas using PCA. The KMO test was proposed by Kaiser, Meyer, and Olkin to measure the adequacy of the sample, and it was mainly used to test the relative size of the simple correlation coefficient and partial correlation coefficient between original variables. In this experiment, a total of 175 soil samples were collected, which had met the requirements for the number of samples for the KMO test, and the data were analyzed and evaluated through the KMO test.

**Figure 10:**
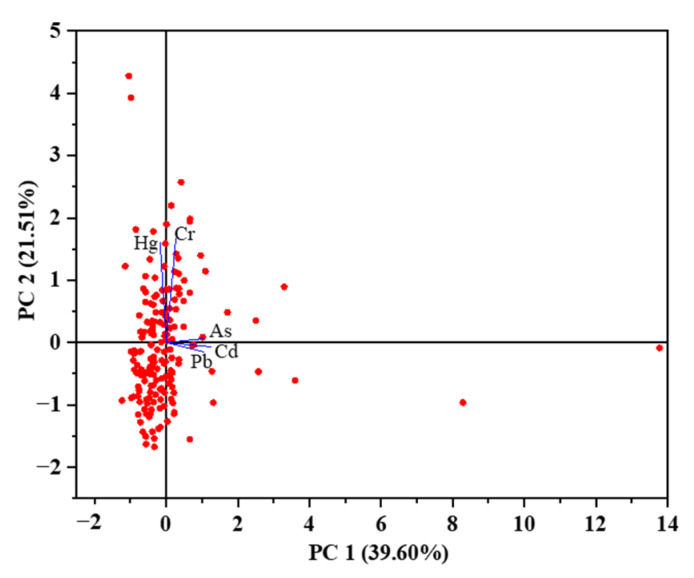
Principal component score plots of five heavy metals.

In this study, the KMO value (0.579 *>* 0.5) and Bartlett’s test (*p* < 0.001) showed that there was a correlation between heavy metal elements, so it was feasible for PCA to be used to analyze the source of heavy metals in the study soil sample. It could be seen from Table 6 that the cumulative variance contribution rate of the first four principal components reached 93.47%, indicating that the first four principal components could reflect most of the information of the data to a certain extent. Moreover, the factor loadings of the two principal components (PC1 and PC2) account for 61.11% of the total variance. PC1 explained 39.60% of the total variance, and the loading coefficients of Cd, Pb, and As were 0.6342, 0.5411, and 0.5276, respectively. PC2 explained 21.51% of the total variance, and the loading coefficients of Cr and Hg were 0.7184 and 0.6916, respectively. The PCA results identified that Cd, Pb, and As could be classified into one group due to their close relationship. Nevertheless, the other two heavy metals (Hg and Cr) were classified into the independent group due to the long relationship distance (See Figure 10).

Besides, Hg and Cr constituted the primary control factors of PC3, whereas the factor load amounts to 0.6, accounting for 19.61% of the total variance. The closely related indexes for PC4 include heavy metals Pb and As, and their factor amount to −0.5006 and 0.7152, respectively. Overall, these results revealed that there were two main sources of heavy metals in bamboo shoots soil from the research areas.

Based on the above results, Pb, Cd, and As were the main pollution factors of heavy metals in bamboo shoot soil of the study area. For the soil of agricultural and forestry producing areas, Pb was generally mainly attributable to transportation emissions, accounting for about 2/3 of global Pb pollution; other anthropogenic activities such as the cement industry may also lead to Pb pollution [61,62,63]. In addition, during the cultivation of agricultural and forestry products, the application of phosphate fertilizers and pesticides may provide a large amount of Cd to planting area soils. Since Cd was an inherent component of phosphate rock, and it was easily transferred to phosphate fertilizers, it further indicates that the application of chemical fertilizers, especially phosphate fertilizers, is an important cause of Cd pollution in soils [64,65]. As in soil might be related to agricultural and forestry activities, such as pesticide application. Environmental heterogeneity due to geographic isolation might account for high arsenic levels in individual soils [66,67]. Besides, Cr contamination might come from the metallurgical industry [68]. Hg pollution was generally believed to be mainly due to human activities. About 40% of the external mercury input to soils in China comes from coal combustion, accounting for more than 70% of the total Hg input to the atmosphere, and the high volatility of Hg makes it easy to enter the flue gas, eventually causing Hg to deposit in the soil through the atmosphere and accumulate [64,69,70,71]. The concentration of Hg in this study area was much lower than the standard limit value, indicating that these factors had relatively little effect on the soil of bamboo shoots. In summary, based on experimental results and relevant source analysis, it could be speculated that the heavy metal pollution sources in the soil of bamboo shoots in Guangdong were mainly the use of fertilizers and pesticides, traffic emissions, and human activities.

## 4. Conclusions

This study included the content investigation, pollution risk assessment, and source analysis of soil heavy metal for bamboo shoot producing areas in Guangdong Province. The results showed that the bamboo shoot soil in the study area was acidic or weakly alkaline. The average contents of Pb (PT-02, JT, FLX-03, HG-02, and HST-03), Cd (HG-02 and HST-03), and As (HST-01 and HST-03) exceeded the risk screening value in some areas. The CV analysis results reflected that in the PT-03 (As), HG-02 (Pb, Cd), HST-01 (Cd), HST-02 (Cd), HST-03 (Pb, Cd and As), and ZA (Cd) areas, the CV values of the corresponding heavy metals had a strong variability. The analysis results of *P_N_* showed that the proportion of soil samples classified as no, slight, and medium accumulation were 67.37%, 28.26%, and 4.35%, respectively, and the values of HG-02 and HST-03 were higher than 2.0. The analysis results of *I_geo_*, $E_{r}^{i}$, and RI illustrated that the soil of the study area could be divided into three pollution grade. The average contribution rates of heavy metals were as follows: Cd > As > Hg > Pb > Cr. In addition, the correlation analysis indicated that pH and Pb (0.193), Pb and Cd (0.153), and Cd and Hg (−0.173) were significantly correlated at the 0.05 level. Particularly, there were extremely significant correlations between pH and Cd (0.221), pH and Hg (−0.277), Pb and As (−0.283), and As and Cr (0.206). PCA results revealed that PC1 accounts for 39.60% of the total variance, which included Cd, Pb, and As; PC2 mainly includes Hg and Cr, accounting for 21.51% of the total variance. The finding suggested that there were differences in the soil pollution grade of Guangdong bamboo shoot production areas, and the main reasons for this result include: the use of fertilizers and pesticides, traffic emissions, and human activities. Therefore, understanding the distribution of heavy metals in soil and conducting a pollution risk assessment are of great significance for preventing soil pollution and ensuring food safety in the bamboo shoots producing area.

## Figures and Tables

**Table 1:** The limit standard for heavy metals in soil.

Indices	pH	Content (mg/kg)				
		Pb	Cd	As	Hg	Cr
Soil environmental background value in China	-	23.5	0.079	9.6	0.038	57.3
Soil environment background value in Guangdong Province (median value)	4.8	34.38	0.034	10.50	0.075	43.25
Minimum (n = 175)	4.07	11.0	0.01	1.0	0.04	4.0
Maximum (n = 175)	8.14	456	2.45	155.24	0.73	175
Mean (n = 175)	4.89	48.91	0.11	18.64	0.13	57.73
pH ≤ 5.5	-	70	0.3	40	1.3	150
5.5 < pH ≤ 6.5	-	90	0.3	40	1.8	150
6.5 < pH ≤ 7.5	-	120	0.3	30	2.4	200
pH > 7.5	-	170	0.6	25	3.4	250

**Table 2:** Coefficient of variation of heavy metal content in different areas.

Area	CV%					
	pH	Pb	Cd	As	Hg	Cr
LK	27.7792	80.8122	3.2889	9.5699	0.0000	0.0000
HS	1.8144	26.9336	0.0000	7.2922	28.2160	34.0818
NJ	3.3864	13.6735	0.0000	57.3032	79.3443	10.5522
PT-01	3.1208	23.7097	36.3308	60.0251	62.6881	53.3241
PT-02	4.2855	1.4560	54.6082	32.8555	65.0904	3.8222
PT-03	12.5026	65.3849	54.4681	121.0049	43.6856	45.9568
PT-04	4.9990	24.0241	70.5584	50.3507	15.9114	37.6806
PT-05	4.3064	94.0409	38.2031	99.7303	76.1120	68.7879
FX	0.0000	3.5355	62.8539	9.9593	0.0000	23.5702
CK-01	2.2222	34.8216	12.3853	34.6369	52.3643	34.1387
CK-02	5.6886	27.1399	21.6506	10.6227	20.4983	31.1265
CK-03	6.6083	14.3970	51.0629	19.2835	76.8646	42.1174
CJ	4.1667	5.2735	69.2820	24.4099	23.5932	35.1001
MZF-01	5.4890	75.2727	58.2323	9.7160	20.4275	31.6118
MZF-02	8.4765	24.4677	57.3285	35.7951	0.0000	22.6866
LH-01	16.6844	5.1992	36.4642	48.0365	34.1563	66.0908
LH-02	6.7924	38.2910	59.6131	31.0259	0.0000	59.2684
JT	3.4139	5.5442	21.5013	11.8720	19.6574	19.9982
XN-01	25.1701	66.8107	20.4050	4.1254	28.6598	17.6253
XN-02	8.1490	76.0044	62.0690	44.9355	28.7052	27.1219
XN-03	16.7080	52.5292	72.3323	44.7120	30.7443	13.0529
XN-04	13.2901	57.9295	74.2816	34.8771	58.8432	33.0903
XN-05	11.4313	81.1947	119.2555	33.7977	0.0000	60.6522
CJ	1.0567	11.8084	27.1522	26.3777	17.2927	11.1770
LJ-01	12.6225	13.1549	96.8644	20.6447	30.8377	8.8168
LJ-02	3.1625	26.7418	60.4706	58.8827	42.8112	9.8201
LJ-03	3.1236	51.3490	30.4290	54.5609	20.7688	16.0180
LJ-04	1.7778	39.5246	91.4814	26.8515	24.1834	22.0949
LJ-05	5.1648	28.1548	47.0748	45.0695	54.3124	17.6202
LJ-06	3.0744	6.8272	0.0000	27.8995	0.0000	1.5639
LJ-07	0.0000	11.7460	0.0000	17.8086	30.6503	10.0274
LJ-08	4.7854	38.4764	64.7585	28.9824	48.7014	13.1076
FLX-01	7.9749	23.0755	22.8218	17.6951	12.7629	20.0386
FLX-02	7.4276	0.9752	47.1405	0.0000	9.4281	0.0000
FLX-03	11.6942	4.1938	60.6092	84.0496	91.9239	0.0000
FLX-04	6.8594	23.0997	47.1405	59.7929	18.4463	37.3877
HG-01	5.6505	27.1314	24.7436	32.0179	39.1361	38.0823
HG-02	2.8862	131.6599	128.5649	23.0328	59.6749	32.0308
HG-03	7.9450	15.9028	0.0000	19.6629	7.4996	32.4108
HG-04	3.0090	7.3856	20.5115	24.5698	1.5316	10.4788
HST-01	11.9869	31.6744	118.0194	79.9434	53.0047	3.8204
HST-02	35.9992	43.2192	139.7405	6.9512	33.1259	20.7521
HST-03	12.6792	125.8060	165.9978	123.3858	13.8564	33.7225
AT	18.7262	42.7932	82.5227	19.7973	47.1265	64.5234
NQ	1.2926	30.1648	40.5636	54.7615	37.0910	22.8291
ZA	3.2548	37.5576	117.0615	59.4019	17.3719	60.7335

**Table 3:** The classification of the contamination degree according to the value of *P_i_* and *P_N_*.

Level	*P_i_*	*P_N_*	Contamination Assessment
1	*P_i_* ≤ 1	*P_N_* ≤ 0.7	No
2	1 < *P_i_* ≤ 2	0.7 < *P_N_* ≤ 1	Slight
3	2 < *P_i_* ≤ 3	1 < *P_N_* ≤ 2	Mild
4	3 < *P_i_* ≤ 5	2 < *P_N_* ≤ 3	Medium
5	*P_i_* > 5	*P_N_* > 3	Serious

**Table 4:** Classification standard for ecological risk assessment of heavy metals in soil.

Classification Standard	Pollution Level				
	Light	Medium	Severe	Intensity	Strong
$E_{r}^{i}$	<30	30–60	60–120	120–240	≥240
RI	<60	60–120	120–240	240–480	≥480

**Table 5:** The $E_{r}^{i}$ and RI levels of five metals in bamboo shoots soil of the research areas.

Area	$E_{r}^{i}$					RI
	Pb	Cd	As	Hg	Cr	
LK	5.0000	21.5000	1.6625	1.5385	0.2133	29.9143
HS	1.8036	5.0000	1.9063	17.5385	1.1633	27.4116
NJ	2.2286	5.0000	0.5850	3.8154	1.2960	12.9250
PT-01	4.4071	7.6150	4.2021	3.7821	0.7356	20.7418
PT-02	5.2036	7.0700	3.7125	3.3292	0.7400	20.0553
PT-03	5.1060	6.2267	6.0796	3.5697	1.1222	22.1042
PT-04	4.1036	9.4433	3.3038	2.6456	0.7800	20.2763
PT-05	3.4959	5.3186	6.2500	3.6286	0.6371	19.3302
FX	2.8571	9.0000	1.7750	4.0000	0.2400	17.8721
CK-01	3.7619	12.3333	2.6500	8.8205	0.4133	27.9791
CK-02	3.7000	5.3333	1.4500	6.2872	0.2267	16.9972
CK-03	4.7095	15.8333	1.6125	2.8718	1.2638	26.2909
CJ	4.5571	8.3333	3.2667	6.5641	0.9973	23.7186
MZF-01	4.4286	8.5000	1.6375	2.7692	1.1333	18.4686
MZF-02	4.5476	7.8333	2.1292	1.5385	1.4556	17.5041
LH-01	3.4429	6.3333	1.5558	5.6308	0.2222	17.1850
LH-02	4.1357	11.0000	0.9500	1.5385	0.3093	17.9335
JT	5.8095	16.3333	1.6083	4.2359	0.3289	28.3160
XN-01	2.0238	12.3333	6.8917	2.3692	0.4622	24.0803
XN-02	2.3393	7.2500	5.2500	2.8615	0.4800	18.1808
XN-03	1.3750	13.7500	5.2813	2.7462	0.5167	23.6691
XN-04	2.4107	21.0000	4.2125	2.7923	0.5833	30.9989
XN-05	1.7223	17.6000	6.4813	1.5385	0.6075	27.9495
CJ	4.0429	7.6667	2.7000	4.3487	0.1822	18.9405
LJ-01	2.0809	9.0000	8.0094	4.2538	1.0560	24.4001
LJ-02	2.8524	6.0200	10.8355	4.4308	1.1110	25.2497
LJ-03	2.0102	6.0000	8.1425	3.4923	0.9006	20.5456
LJ-04	2.9548	5.2667	8.8333	4.5026	0.9551	22.5124
LJ-05	3.0536	20.6500	4.5188	7.9615	1.0243	37.2082
LJ-06	2.5893	5.0000	3.6750	1.5385	0.8440	13.6467
LJ-07	2.1500	5.0000	5.0625	4.0462	1.0907	17.3493
LJ-08	2.1321	11.1500	9.4500	4.0615	1.0787	27.8723
FLX-01	3.7643	2.4000	2.8550	5.6985	0.3280	15.0457
FLX-02	4.9204	3.0000	0.5000	2.3077	0.1067	10.8347
FLX-03	6.4793	3.5000	1.2325	6.1538	0.1067	17.4723
FLX-04	2.3614	3.0000	0.8663	3.5385	0.4569	10.2231
HG-01	2.1286	2.3333	6.7000	6.2256	0.9911	18.3787
HG-02	16.8679	55.0000	7.6750	2.6615	0.8153	83.0197
HG-03	1.7786	5.0000	4.6750	4.0615	1.1607	16.6758
HG-04	1.8464	13.1000	9.1375	4.2615	1.1247	29.4701
HST-01	2.8121	14.0000	10.9275	3.1590	0.8090	31.7076
HST-02	1.6733	23.0000	4.8092	3.8256	0.8940	34.2021
HST-03	8.0298	84.0000	16.0092	2.5641	0.8789	111.4819
AT	3.8774	6.6667	1.6921	4.0051	0.1400	16.3813
NQ	3.3571	9.3333	1.6250	2.6667	1.0267	18.0088
ZA	1.2214	8.7500	2.6288	1.6615	1.2267	15.4884
Minimum (n = 46)	1.2214	2.3333	0.5000	1.5385	0.1067	10.2231
Maximum (n = 46)	16.8679	84.0000	16.0092	17.5385	1.4556	111.4819
Mean (n = 46)	3.6555	11.9299	4.5003	4.0834	0.7442	24.9134
Standard deviation	2.4661	13.8323	3.3598	2.6142	0.3810	17.0144
Sum	168.1535	548.7769	207.0132	187.8383	34.2356	1146.0176

**Table 6:** Eigenvalues, variance contribution rates, cumulative contribution rates, and component matrix of the principal components of heavy metals.

Item	PC1	PC2	PC3	PC4
Eigenvalues	1.9798	1.0753	0.9807	0.6373
Variance contribution rates	39.60	21.51	19.61	12.75
Cumulative contribution rates	39.60	61.11	80.72	93.47
Pb	0.5411	−0.0636	0.4072	−0.5006
Cd	0.6342	−0.0286	0.1047	−0.0435
As	0.5276	0.0280	−0.2866	0.7152
Hg	−0.0884	0.6916	0.6453	0.3109
Cr	0.1377	0.7184	−0.5698	−0.3733

## Data Availability

The datasets generated for this study are available on request to the corresponding author.
